# Ochratoxin A in Poultry Supply Chain: Overview of Feed Occurrence, Carry-Over, and Pathognomonic Lesions in Target Organs to Promote Food Safety

**DOI:** 10.3390/toxins16110487

**Published:** 2024-11-10

**Authors:** Elisabetta Bonerba, Alessio Manfredi, Michela Maria Dimuccio, Patrizio Lorusso, Annamaria Pandiscia, Valentina Terio, Angela Di Pinto, Sara Panseri, Edmondo Ceci, Giancarlo Bozzo

**Affiliations:** 1 Department of Veterinary Medicine, University of Bari Aldo Moro, Provincial Road to Casamassima km 3, 70100 Valenzano, Italy; elisabetta.bonerba@uniba.it (E.B.); michela.dimuccio@uniba.it (M.M.D.); patrizio.lorusso@uniba.it (P.L.); annamaria.pandiscia@uniba.it (A.P.); valentina.terio@uniba.it (V.T.); angela.dipinto@uniba.it (A.D.P.); edmondo.ceci@uniba.it (E.C.); giancarlo.bozzo@uniba.it (G.B.); 2 Department of Veterinary Medicine and Animal Science, University of Milan, Via dell’ Università 6, 26900 Lodi, Italy; sara.panseri@unimi.it

**Keywords:** OTA, human hazard, feed, poultry products, food safety, histopathology

## Abstract

Ochratoxin A (OTA) is a mycotoxin produced by fungi species belonging to the genera *Aspergillus* spp. and *Penicillium* spp. The proliferation of OTA-producing fungal species may occur due to inadequate practices during both the pre-harvest and post-harvest stages of feed. Consequently, poultry species may be exposed to high concentrations of this mycotoxin that can be transferred to animal tissues due to its carry-over, reaching dangerous concentrations in meat and meat products. Therefore, this review aims to propose a comprehensive overview of the effects of OTA on human health, along with data from global studies on the prevalence and concentrations of this mycotoxin in avian feeds, as well as in poultry meat, edible offal, and eggs. Moreover, the review examines significant gross and histopathological lesions in the kidneys and livers of poultry linked to OTA exposure. Finally, the key methods for OTA prevention and decontamination of feed are described.

## 1. Introduction

Ochratoxins are a group of mycotoxins mainly produced by fungi belonging to the genera *Aspergillus* spp. and *Penicillium* spp. [1]. Based on their chemical structures, they can be classified into three different types: ochratoxin A (OTA), ochratoxin B (OTB), and ochratoxin C (OTC). Among them, OTA occurs with a high frequency in food stuffs and is the most toxic [1,2,3]. Several studies proved that OTA has nephrotoxic, immunotoxic, hepatotoxic, teratogenic, and carcinogenic effects in many animal species [4,5,6,7]. In 1993, the International Agency for Research on Cancer classified OTA as a potential human carcinogen (group 2B) due to extensive evidence of its carcinogenicity found in multiple animal studies [8]. Therefore, to safeguard the health of European Union (EU) consumers, especially the most vulnerable consumer groups such as children and pregnant women, the European Commission laid down maximum levels for OTA in foods [9].

OTA contamination can occur in several products that are widely prevalent in human and animal diets because of their susceptibility to colonization by molds. These are cereal, coffee, vegetable, licorice, raisins, wine, dried fruits, spices, legumes, oil seeds, pork, and poultry products [10,11]. After oral ingestion, OTA is rapidly absorbed and through enterohepatic circulation reaches high levels in the blood within a few hours in many animal species [12]. The bioavailability of OTA is around 50%, with 66% in pigs, 56% in rats and rabbits, and 40% in chickens, and it is largely influenced by the species, dose, vehicle, and food in the stomach during OTA administration [12]. In addition, a very important feature of this mycotoxin is the ability to bind serum proteins, such as albumin, causing a delay in its elimination from blood and excretion, increasing its half-life in the animal body [12]. Considering the OTA’s high half-life, it can be transferred to animal tissues due to its carry-over, reaching dangerous levels in meat and meat products [12,13,14].

Among food-producing animals, poultry is one of the most susceptible to OTA contamination. Indeed, different studies showed that maize, a major component of poultry feed, can be contaminated with OTA, especially during handling and storage [15,16]. After ingestion, OTA is capable of both altering the tight junctions in the intestinal epithelium and the local immune response, inducing a systemic condition known as chronic mycotoxicosis associated with several organs’ damage [17]. For example, pathophysiological changes such as altered proximal tubular function, ultrastructural degeneration of renal integrity, reduced glomerular filtration rate, and reduced urine concentration have been documented at the renal level [14]. Similarly, adverse necrosis and hemorrhages have been recorded in the liver [14]. Furthermore, OTA in poultry can accumulate both at the level of the muscle fibrils and at the level of the ovary, inducing during the embryonic phase extensive oxidative stress and organ damage due to its carry-over into the eggs [18]. All these alterations induce increased weakness, decreased feed consumption, and reduced growth rate and egg production in poultry. In addition, increased mortality is recorded in breeding [19].

Therefore, although the presence of OTA within the edible tissues of poultry species is well documented, in Europe, the Commission Regulation (EU) 2023/915 of 25 April 2023 on maximum levels for certain contaminants in food does not establish maximum OTA levels in poultry meat and poultry-derived products [9]. A few European countries such as Denmark, Estonia, Slovakia, Romania, and Italy have adopted guidelines on the concentration of OTA in meat and/or meat products [20,21,22]. In Canada, Asia, the USA, and Australia, on the other hand, no maximum limits are set for the presence of this mycotoxin in these products [20,23]. Regarding feed intended for food-producing animals, the European Commission Recommendation (EU) 2016/1319 set the guidance value of 0.25 mg kg^−1^ for cereals and cereal products for feed materials [24]. On the other hand, guidance values of 0.1 mg kg^−1^ and 0.05 mg kg^−1^, respectively, are set for compound feed intended for poultry and pigs [24].

With those premises, this study aims to describe the state of the art related to the implications of OTA on human health, the prevalence of OTA in food and feed with a focus on poultry species, and the occurrence of OTA related with gross and histopathological lesions observed in poultry kidneys and livers. Finally, the main methods for OTA prevention and decontamination of feed are described.

## 2. Human Hazard

The relationship between OTA exposure and human health risk is still a hotly debated topic. In fact, although the classification of this mycotoxin by the International Agency for Research on Cancer (IARC) as being possibly carcinogenic to humans is still in effect, the European Food Safety Authority (EFSA) considered during the last risk assessment (2020) that it was not appropriate to maintain a Health-Based Guidance Value (HBGV), making the tolerable weekly dose of 120 mg kg^−1^ established in 2006 by the EFSA Panel on Contaminants in the Food Chain invalid, as the molecular mechanisms underlying the genotoxic action of OTA in vivo remain unclear [12,25]. Therefore, risk characterization for OTA against defined toxicological reference values for non-neoplastic and neoplastic effects is performed using a parameter known as the Margin of Exposure (MOE). According to the EFSA guidelines for non-neoplastic and neoplastic effects, an MOE of 200 and 10,000 or higher, respectively, represent a low health concern. During the latest EFSA surveys for non-neoplastic effects, an MOE of about 200 was calculated for OTA, and for neoplastic risk a value of less than 10,000, identifying high consumers of foods at risk of OTA contamination and breastfed infants as risk groups. In addition, for the characterization of non-neoplastic effects, a Benchmark Dose Lower bound 10% (BMDL10) equal to 4.73 µg kg^−1^ body weight per day was identified. In contrast, for the characterization of neoplastic effects, a BMDL10 of 14.5 µg kg^−1^ body weight per day was calculated [12].

In general, although ochratoxin can cause toxicity in several organs, the main target organ of this mycotoxin is the kidney. Indeed, it is recognized as a potent nephrotoxin, due to its ability to accumulate in proximal tubule epithelial cells and induce cell damage through oxidative stress, DNA damage, apoptosis, pyroptosis, and cell cycle arrest [7,26,27].

Oxygen free radicals (ROS) such as hydrogen peroxide, hydroxyl radical, and superoxide anion play an important role in cellular homeostasis; however, when their levels increase, they can generate oxidative stress by promoting cell death, mutations, chromosome aberrations, and carcinogenesis [28]. Nowadays, different studies are evaluating the role of oxidative stress in OTA-mediated renal toxicity [29,30,31,32]. García-Pérez et al. in their study observed how OTA has a cytotoxic effect on human renal proximal tubule cells (HK-2) that occurs with an increase in the presence of ROS and a decrease in glutathione [33]. Similarly, Lee et al. showed that treatment of HK-2 cells treated with OTA increased intracellular reactive oxygen species and malondialdehyde and decreased glutathione levels simultaneously with an increase in indicators of kidney damage [34]. All this evidence suggests a central role of oxidative stress in OTA-induced renal cytotoxicity [33].

One event dependent on increased oxidative stress induced by OTA is DNA damage. In fact, numerous studies have shown that this mycotoxin is not capable of direct genotoxicity in renal cells and kidneys but indirectly through ROS production and oxidative stress induction [35,36]. Recently, Girgis et al. showed how OTA-treated cells went into increased ROS production leading to both structural and numerical chromosome alterations and DNA damage compared with untreated controls [37]. Moreover, continuous exposure of proximal tubular epithelial cells to OTA over time resulted in the formation of micronuclei inducing karyomegaly and chromosomal instability prior to the formation of proliferative lesions [38].

Another event related to increased ROS production is apoptosis, which is a type of programmed death related to caspase family genes and Bcl-2 in mitochondria-mediated apoptosis. In the bcl-2 family, Bax is a pro-apoptotic protein. It determines the release of cytochrome c by promoting the cascade of caspases leading to apoptosis. In contrast, Bcl-2 is an anti-apoptotic protein as it inhibits the release of cytochrome C by mitochondria [39,40,41,42]. Song Y et al. showed that exposure of the HK-2 cells to Ochratoxin A induces apoptosis in a manner directly proportional to the concentration of OTA, inducing an increase in the expression of caspase-3 and Bax, with a concomitant decrease in the expression of Bcl-2 [43]. In addition, OTA on human renal proximal tubule cells induces apoptosis by activation of the NF-kb pathway with increased phosphorylation of ERK-1/2 and by activation of the JNK apoptotic pathways related to endoplasmic reticulum stress and cleavage of caspase precursor 4 due to an increase in ROS [44,45].

Pyroptosis is a recently discovered mechanism of pro-inflammatory programmed necrosis. The canonical process is mediated by the cleavage of gasdermin D (GSDMD) by activated caspase-1 into N-terminal and C-terminal fragments [46,47]. Subsequently, the N-terminal fragment translocates across the plasma membrane and causes its perforation, allowing the release of pro-inflammatory cytokines such as IL-1β and IL-18, generating osmotic swelling followed by cytolysis [48,49]. Instead, the noncanonical process is mediated by lipopolysaccharide that activates caspase-4/5/11 inflammasome that, cleaving GSDMD, induces pore formation on the cell membrane and pyroptosis [50]. OTA has been shown to induce this cell death mechanism in both in vivo and in vitro models. Male C57BL/6 mice were injected intraperitoneally with OTA at a concentration of 2 mg kg^−1^ body weight resulting in an increase in renal fibrosis-related molecules (α-SMA, Vimentin, TGF-β) that activate the inflammasome NOD-like receptor 3 (NLRP3), inducing pyroptosis at the renal level. In addition, exposure of Madin–Darby canine kidney epithelial cells to 2 μg mL^−1^ of OTA showed similar effects characterized by NLRP3 inflammasome activation and pro-inflammatory cytokine release [51,52].

Finally, an additional mechanism of OTA-induced nephrotoxicity could be related to the role of this mycotoxin in epigenetic processes such as DNA methylation and histone modifications which are deeply involved in several diseases [53]. In this regard, it has recently been shown that OTA is able to induce changes in DNA methylation in human renal proximal tubular cell lines by leading to cell cycle arrest in the G0/G1 phase, suggesting how this mechanism may be involved in carcinogenesis [54].

In addition to experimental studies carried out in in vitro and in animal models, the effects that OTA has on human health have been inferred from epidemiological studies. OTA is associated with a condition known as Balkan endemic nephropathy (BEN), which is endemic in Balkan areas such as Bosnia and Herzegovina, Croatia, Bulgaria, and Romania [55]. The clinical symptoms of this condition are exhaustion, pallor of the skin, lower back pain, anemia, proteinuria, and uremia. Furthermore, the pathological symptoms are similar to tubulo-interstitial diseases of the kidney and are mainly characterized by progressive atrophy and sclerosis of all structures of the kidney [56]. The association between BEN and OTA arises mainly from the high levels of this mycotoxin found in human plasma and urine analyzed in regions where this disease is endemic and because of the similarity of the symptoms of the disease with porcine nephropathy induced by OTA [55,57]. In addition, other epidemiological studies have evaluated the association between blood OTA levels with other kidney diseases such as chronic interstitial nephropathy, chronic glomerular nephropathy, and chronic vascular nephropathy [58,59].

Therefore, despite the presence of the extensive literature on the in vitro and in vivo effects of OTA, the evaluation of the in-human effects is still being studied today.

## 3. OTA in Food and Feed

### 3.1. Occurrence of OTA in Feed and Transmission to Avian Species

Feeds used for avian species are characterized by a mix of grains such as maize, wheat, sorghum, and rice [60]. They also include animal proteins and plant proteins [18]. These types of feeds are very susceptible to fungal growth especially if stored incorrectly, for example, at too hot temperatures or high humidity. These conditions create an ideal environment for the proliferation of *Aspergillus* spp. and *Penicillium* spp. capable of producing OTA [60].

Several studies around the world have evaluated the level of OTA contamination of avian feeds [60,61,62]. For example, Sherazi et al. evaluated the occurrence of OTA in 80 complete poultry feeds and 286 poultry feed ingredients through an indirect enzyme immunoassay and high-performance liquid chromatography coupled with fluorescence detection (HPLC-FD). The frequency of contamination and the mean OTA levels for the ingredients were 31% and 51 μg kg^−1^, respectively. In particular, the most contaminated ingredient was maize with a mean OTA value of 80.8 μg kg^−1^. On the other hand, for complete feeds, the corresponding values were 38% and 75 μg kg^−1^. The guidance value of 0.1 mg kg^−1^ was exceeded by the OTA levels in ten samples of complete poultry feed and 19 samples of feed ingredients [60].

The importance in monitoring the occurrence of this mycotoxin within feed is mainly due to the concern related to the possible transmission of OTA in poultry and the subsequent distribution in the edible parts of these species (Table 1). In this regard, Pozzo et al. performed an experiment in which 36 one-day-old male broiler chicks were fed with feed supplemented with 0.1 mg OTA kg^−1^ [63]. Then, the concentration of OTA was evaluated in serum, liver, kidney, breast, and thigh samples. The mycotoxin was detected in the serum, liver, and kidney at a concentration of 1.2 ± 0.4 ng mL^−1^, 1.9 ± 0.2 μg kg^−1^, and 3.6 ± 0.9 μg kg^−1^, whereas it was not detectable in breast and thigh [63]. Similar results were described in the study performed by Bozzo et al. in which laying hens, fed with feed having an average OTA concentration of 255 and 285 μg kg^−1^, had OTA at a detectable concentration only in the kidney and liver, but not in all other tissues [10]. However, in the study conducted by Zaghini et al., laying hens fed with 0.2 mg kg^−1^ of OTA added to the basal diet also had a detectable concentration of this mycotoxin in muscles. Specifically, its concentration was highest in the kidney (2.47 ± 1.10 μg kg^−1^) and decreased in the blood (1.06 ± 0.48 μg kg^−1^) and muscle (0.31 ± 0.22 μg kg^−1^) [64]. Similarly, in the study by Birò et al., in broiler chicks exposed to feed contaminated with 0.5 mg kg^−1^ OTA was distributed as follows: liver > kidney > plasma > muscle [65].

Therefore, all these studies show that OTA accumulated mainly in the kidney and liver. The main reason for this observation was that this mycotoxin was eliminated chiefly by hepatic and renal via [66]. Moreover, the active role of the organic anion transporter polypeptides has recently been recognized. Indeed, they can mediate the uptake of OTA at the hepatic and renal levels by active absorption (basolateral expression) or in tubular reabsorption (apical expression), explaining the accumulation and related toxicity exerted mainly on these organs [66].

### 3.2. Occurrence of OTA in Meat and Edible Offal in Avian Species

Data obtained on the occurrence of OTA in poultry edible tissues from studies carried out worldwide are summarized in Table 2.

In Europe, one of the first studies on the presence of OTA in avians was carried out in Denmark by Jørgensen et al. Samples of duck (liver and muscle), turkey (liver and muscle), goose (liver and muscle), and chicken (muscle) were analyzed via HPLC. In general, the concentration of OTA measured for each sample was low; the highest levels of this mycotoxin were found in turkey liver (0.28 μg kg^−1^). On the other hand, the highest prevalence of OTA was recorded for turkey meat samples (10/17, 58.8%) [67]. Similar results were reached by Guillamont et al. and Guerrini et al. In the first case, a measurable concentration of OTA was found only in turkey muscle [68]. In the second case, kidney samples from backyard chickens were all negative for OTA, suggesting that this category of poultry is less exposed to contamination from this mycotoxin [69]. Moderate levels of contamination of chicken samples were described by Milićević et al. with concentration levels ranging from 0.14 to 3.9 µg kg^−1^ in the livers, 0.1 to 7.02 µg kg^−1^ in the kidneys, and 0.25 to 9.94 µg kg^−1^ in the chicken gizzards [70]. Finally, in a recent study, Bozzo et al. found higher levels of this mycotoxin in the liver and kidney of laying hens, with average OTA levels of 24 ± 1.92 µg kg^−1^ and 47 ± 3.03 µg kg^−1^, respectively [14].

In Asia, as in Europe, several studies have been carried out. Iqbal et al. conducted a study in Pakistan measuring OTA levels in 115 meat samples from chicken broiler, chicken layers, and domestic chickens, including the liver. The prevalence of this mycotoxin was 40.8%, and the samples with the highest concentration were chicken layers’ livers with an average OTA value of 2.41 ± 0.72 µg kg^−1^ [71]. Furthermore, in agreement with the study conducted by Guerrini et al., backyard chickens also did not show traces of OTA, strengthening the hypothesis that this category of avian is little exposed to this type of contamination [69,71]. In Jordan, on the other hand, two studies were conducted by AL khalail and Alaboudi et al., respectively. In the first case, 100% of the thigh and leg, liver, gizzard, and 66.6% of poultry breast samples were positive for OTA; moreover, in agreement with the study by Iqbal et al., the samples with the highest average OTA concentration were the chicken liver samples (5.860 ± 0.390 µg kg^−1^) [72]. In the second study, however, samples of chicken meat, liver and kidney analyzed using liquid chromatography/time-of-flight/tandem mass spectrometry were all negative for the presence of OTA [73]. Similarly, low contamination values of this mycotoxin were found in chicken liver samples analyzed by Cao et al. in China with an average OTA level of 1.05 µg kg^−1^ [74]. Finally, in the study conducted in Iraq by Murad, very high prevalence levels of OTA were calculated in samples of chicken meat and the liver (86.7% and 57%); however, the average measured concentration was moderate (1.982 µg kg^−1^ for chicken meat samples and 1.865 µg kg^−1^ for chicken liver samples) [75].

In Africa, two studies were performed by Tatfo et al. in Cameroon and by Elgazzar in Egypt. In the first one, samples of chicken muscle and liver were analyzed. In line with the other studies, the highest levels of OTA were found in the liver with a mean value of 2.27 ± 1 µg kg^−1^ versus 1.4 ± 0.173 µg kg^−1^ measured in the muscle [76], whereas in the second study, muscle, fat, gizzard, kidney, and liver samples from broilers (50 and 100 days old) and hens (2 years old) were analyzed. The highest average OTA value was found in the hen kidney (18.84 ± 3.30 µg kg^−1^), while the lowest value was measured in broiler muscle (5.94 ± 2.21 µg kg^−1^) [77].

Therefore, although studies conducted to monitor the presence of OTA in the tissues of animals raised on poultry farms are rather limited, the above data generally show non-negligible levels of positivity, demonstrating that poultry exposure to this mycotoxin is quite common in many countries around the world.

### 3.3. Occurrence of OTA in Eggs

Another concern related to the presence of OTA in avian species is the possible carry-over of this mycotoxin throughout the chain production of eggs. In this regard, several studies assessed the concentration of OTA in eggs after the exposure of birds to diets contaminated with this mycotoxin (Table 3). Bozzo et al. measured OTA levels in eggs produced by laying hens exposed for 30 days to diets contaminated with different concentrations of OTA: 100 μg kg^−1^ (Commission Recommendation (EU) 2016/1319 guidance value); 200 μg kg^−1^; 1000 μg kg^−1^; and 2000 μg kg^−1^. In all groups and controls, Ochratoxin A was not detected in the eggs [78]. Similar results were described by Denli et al. Twenty-eight laying hens exposed to a diet contaminated with a concentration of 2000 μg kg^−1^ OTA for 3 weeks produced negative results for the occurrence of this mycotoxin in eggs [79]. Hassan et al., on the other hand, exposed laying hens to diets with OTA concentrations 30 and 50 times higher than the guidance values set by the Commission Recommendation (EU) 2016/1319 (i.e., 100 μg kg^−1^), finding OTA values in eggs of 4.857 ± 0.23 μg kg^−1^ after 13 days of exposure to a concentration of 3000 μg kg^−1^ and 7.396 ± 1.03 μg kg^−1^ after 21 days of exposure to a concentration of 5000 μg kg^−1^ [80]. Therefore, all these studies show that eggs are a safe food for consumers in terms of OTA contamination. Important concentrations of this mycotoxin are reached after prolonged exposure of avian species to high concentrations of OTA (at least 30 times above the guidance values set by the EU Commission Recommendation).

## 4. OTA-Induced Lesions in Poultry Kidney and Liver

### 4.1. Gross Lesions

Several studies described the effects of OTA on the kidney and liver of poultry species, the main target organs of this mycotoxin. In particular, the gross lesions observed in the kidney are congestion, pallor, and organ swelling [10,78,81,82,83,84,85,86]. Moreover, Bozzo et al. and Hameed et al. showed in laying hens and broiler chicks treated with concentrations of 0.4, 0.8, 1, and 2 mg kg^−1^ of OTA (i) hemorrhagic lesions, (ii) cortical hyperemia, and (iii) presence of radial red streaks, suggesting infiltration of the cortex [78,85]. On the other hand, in the liver, the most described effects were congestion, swelling, and pallor, and some studies reported the presence of hemorrhages [10].

### 4.2. Histopathological Lesions in Kidney

**Figure 1:**
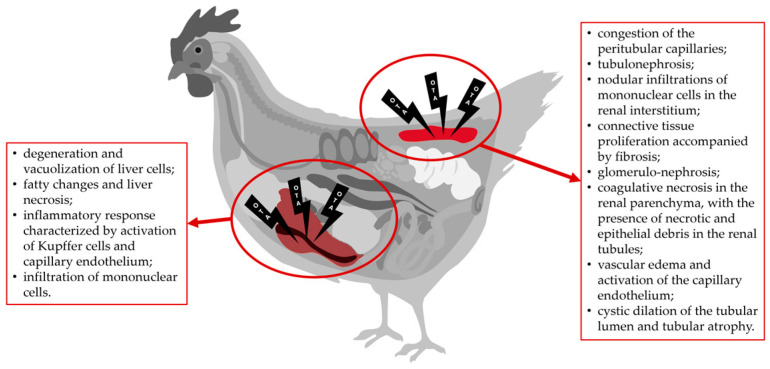
Pathognomonic lesions induced by OTA in poultry kidney and liver.

The most frequently reported histopathological lesions in the kidney are (i) congestion of the peritubular capillaries [81,82]; (ii) tubulonephrosis [78,81,82,83,84,85,87,88,89]; (iii) nodular infiltrations of mononuclear cells in the renal interstitium [65,81,82,84]; (iv) connective tissue proliferation accompanied by fibrosis [81,82,84]; (v) glomerulo-nephrosis [65]; (vi) coagulative necrosis in the renal parenchyma, with the presence of necrotic and epithelial debris in the renal tubules [83,88]; (vii) vascular edemas and activation of the capillary endothelium [81,82,84]; and (viii) cystic dilation of the tubular lumen and tubular atrophy [86] (Figure 1).

Studies carried out by Stoev et al. showed that broiler chicks treated with concentrations of OTA from 0.3 to 5 mg kg^−1^ exhibited congestion of the peritubular capillaries, a sign of altered blood circulation within the kidneys, which can impair renal function [81,82]. This effect was particularly pronounced in groups treated with high doses of OTA and with prolonged exposure [81,82].

Several studies observed significant alterations in the epithelial cells of the proximal convoluted tubules of the kidneys in broilers, turkey poults, and laying hens treated with OTA concentrations ranging from 0.3 to 5 mg kg^−1^. These lesions included cell enlargement, granular and hydropic degeneration, massive necrosis, and vacuolar changes [78,81,82,83,84,87,88]. Pyknotic nuclei, signs of advanced cellular degeneration indicating irreversible cell damage, were reported [10,85,89].

Stoev et al. and Koynarski et al. highlighted nodular infiltrations of mononuclear cells in the renal interstitium in both broiler chicks and turkey poults exposed to OTA concentrations of 0.3, 0.79, 2, and 5 mg kg^−1^ [81,82,84]. Furthermore, Birò et al. showed focal infiltrations of lymphocytes and histiocytes in broiler chicks treated with 0.5 mg kg^−1^ of OTA [65]. These infiltrations indicate an inflammatory response in the renal tissue, which can further impair renal function and lead to permanent tissue damage.

In the kidneys of broiler chicks and turkey poults exposed to high doses of OTA, from 0.3 to 2 mg kg^−1^, connective tissue proliferation accompanied by fibrosis was observed [81,82,84]. The proliferation of connective tissue and fibrosis represent the attempt of the organ to repair damage but can lead to long-term loss of renal function.

Birò et al., in broiler chicks exposed to 0.5 mg kg^−1^ of OTA, described glomerulo-nephrosis characterized by enlarged glomeruli, endothelial, and mesangial cells, resulting in thickening of the glomerular basement membrane in one or more capillary loops [65]. These alterations can impair glomerular filtration, one of the main functions of the kidneys, and lead to renal failure.

Studies by Gupta et al. and Kumar et al. in broiler chicks treated with OTA concentration of 2 mg kg^−1^ reported coagulative necrosis in the renal parenchyma, with the presence of necrotic and epithelial debris in the renal tubules [83,88]. Coagulative necrosis is related to extensive and irreversible tissue damage, while debris in the tubules can obstruct urine flow and further exacerbate renal damage.

Vascular edema and activation of the capillary endothelium were observed in studies carried out by Stoev et al. and Koynarski et al. in broiler chicks and turkey poults treated with 0.3, 0.79, and 2 mg kg^−1^ of OTA [81,82,84]. Edema and capillary proliferation are signs of inflammation and can compromise renal function by increasing internal pressure in the kidneys and causing structural damage.

Finally, Vasiljević et al. described cystic dilation of the tubular lumen and tubular atrophy in the kidneys of laying hens treated with 1 mg kg^−1^ OTA. These structural changes can reduce the ability of kidney to filter blood and produce urine, leading to progressive renal failure [86].

### 4.3. Histopathological Lesions Caused by OTA in Livers

The most documented histopathological lesions in the liver are (i) degeneration and vacuolization of liver cells [10,81,84,87,90]; (ii) fatty changes and liver necrosis [10,63,81,82,88]; (iii) inflammatory response characterized by activation of Kupffer cells and capillary endothelium [81,84,88]; and (iv) infiltration of mononuclear cells [81,83] (Figure 1).

Several studies reported the degeneration and vacuolization of hepatic cells as key effects of avian exposure to OTA. Stoev et al. described ochratoxicosis in broiler chicks exposed to a concentration of 0.79 mg kg^−1^ of OTA showing mild liver enlargement, marked cellular degeneration, and vacuolization [81]. Santin et al. observed vacuolization and megalocytosis of hepatocytes in broilers fed a diet containing 2 mg kg^−1^ of OTA, highlighting a clear morphological lesion of hepatocytes [90]. Koynarski et al. and Hanif et al. described similar histopathological signs in turkey poults and broilers exposed to OTA concentrations of 0.4, 0.8, and 2 mg kg^−1^, respectively [84,87]. Finally, Bozzo et al. observed vacuolated hepatocytes in laying hens exposed to OTA concentrations between 255 and 285 μg kg^−1^, confirming the presence of vacuolar changes even at relatively low doses of OTA [10].

Fatty changes and hepatic necrosis are additional significant manifestations of Ochratoxin A (OTA) intoxication. Stoev et al. reported fatty changes in hepatic cells of broilers treated with OTA concentrations of 0.79 and 5 mg kg^−1^, showing worsening of lesions with prolonged exposures [81,82]. Bozzo et al. observed fatty infiltration of hepatocytes and the presence of clusters of necrotic hepatocytes scattered in the hepatic parenchyma of laying hens fed a diet containing OTA concentrations between 255 and 285 μg kg^−1^ [10]. Gupta et al. identified focal areas of coagulative necrosis, infiltrated by heterophils and mononuclear cells, in broiler chickens treated with 2 mg kg^−1^ of OTA, with increased severity of lesions correlating with higher exposure levels [88]. Pozzo et al. (2013) identified hydropic degeneration with the foci of necrosis in hepatocytes of broilers exposed to 0.1 mg kg^−1^ of OTA [63].

Activation of Kupffer cells and endothelial cells has been observed in various studies, confirming the inflammatory response induced by OTA. This activation was described by Stoev et al. in broiler chicks treated with 0.79 mg kg^−1^ of OTA, by Koynarski et al. in turkey poults exposed to 2 mg kg^−1^ of OTA, and by Gupta et al. in broiler chickens treated with 2 mg kg^−1^ of OTA [81,84,88].

Finally, mononuclear cell infiltration was another frequently documented lesion in OTA exposure studies. Stoev et al. observed the infiltration of perivascular mononuclear cells in broiler chicks treated with 0.79 mg kg^−1^ of OTA, suggesting an acute inflammatory response [81]. Similarly, Kumar et al. identified a mild infiltration of mononuclear cells in portal areas of chicks fed a diet containing 2 mg kg^−1^ of OTA, with increased severity in the presence of bacterial infections [83].

## 5. Prevention and Decontamination Methods from OTA in Feed

As shown in the previous sections, the intake of feed contaminated with OTA affects poultry health and can result in the presence of OTA in foods derived from these species. Moreover, this issue is becoming increasingly important because of climate change which is causing an expansion of fungal occurrence in feedstuffs [18]. Therefore, to reduce fungal and mycotoxin contamination throughout the feed production chain (plant growth, harvest, storage, and distribution), prevention strategies must be implemented at an integrative approach due to multiple possible sources of infection [21]. The intervention should take place prior to any fungal infestation or when mold invades plant material and produces mycotoxins, as well as when agricultural products have been found to be heavily contaminated [21]. Codex Alimentarius has developed codes of practices for the prevention and reduction in OTA contamination in grains used as raw materials for feed composition. Specifically, these include good agricultural and manufacturing practices that need to be applied in the different stages of planting, pre-harvest, harvest, preservation, storage, and transport as well as the use of an OTA management system in the feed supply chain [91].

Different strategies can be applied in pre-harvest and harvest stages to minimize OTA contamination of grain [21].

First of all, at the pre-harvest stage, it has been observed how different genotypes of the same crop species may show different resistances to fungal growth. Therefore, the first strategy applied to control mycotoxin contamination is crop selection, although it is limited by the environment and limited heritability [92]. Crop management has an important impact on the presence of mycotoxins at this stage. This includes proper soil irrigation, soil tillage, crop rotation, proper fertilizer uses, and chemical control or biocontrol of plant diseases [21,92,93]. In addition, prevention from insect attacks is critical due to the ability of insects in interfering with crop resistance to mycotoxin development. In this regard, the use of authorized pesticides (wherever possible) could result in a higher protection against mycotoxin contamination [92]. Finally, forecasting computer models using field parameters and weather inputs are being developed to predict mycotoxin contamination in cereals at the pre-harvest stage [21].

In the post-harvest stage, grain storage is a crucial point to prevent the formation of fungi that can produce OTA [92]. It must be conducted under well-controlled conditions, checking even such minimal details as the adequacy of drainage pipes in the facility where the product is stored and ensuring a high degree of cleanliness between the storage of one crop and the next [94]. This stage is highly dependent on the presence of abiotic and biotic factors [92]. Regarding abiotic factors, an important role is played by temperature and humidity. It is important to control the moisture content and water activity of the grains as well as relative humidity and the temperature during storage [93]. It is necessary to keep products at a water activity of less than 0.7 by storing them in metal containers at a temperature below 20 °C. For unpackaged products, on the other hand, a temperature between 2 and 3 °C is recommended [94]. Among the biotic factors, bacteria (or other microbes) and insects can cause mycotoxin problems in the grain during storage [92]. Specifically, insects compromise biodynamics in the storage ecosystem by producing heat and water metabolites that form “hot spots” within the grain heap. These spots can form an ideal environment for the growth of mycotoxigenic fungi [92].

Therefore, the control of abiotic and biotic factors is necessary to maintain high safety and hygiene of feed.

When the problem of mycotoxins cannot be solved by prevention practices, decontamination methods need to be used. These include chemical methods (such as oxidation, alkalization, hydrolysis, reduction, conjugation, and hydration) and physical methods (such as cleaning and sorting, dehulling milling, adsorption, heating, irradiation, ultrasound, and pulsed light) [92,95].

However, each of these methods shows limitations. Physical treatments are expensive, time consuming, often inefficient, and linked to the loss of large quantities of product [21,95].

Chemical treatments, on the other hand, produce undesirable toxic residues, posing a threat to human health and the environment [95].

For these reasons, chemical decontamination is not legal in the European Union, and the recommended treatment methods, although they have limitations as described above, are the uses of absorbent substances and sorting procedures [21]. Therefore, to overcome the limitations associated with mycotoxin decontamination of feed, new technologies such as cold plasma are being investigated [95].

**Figure 2:**
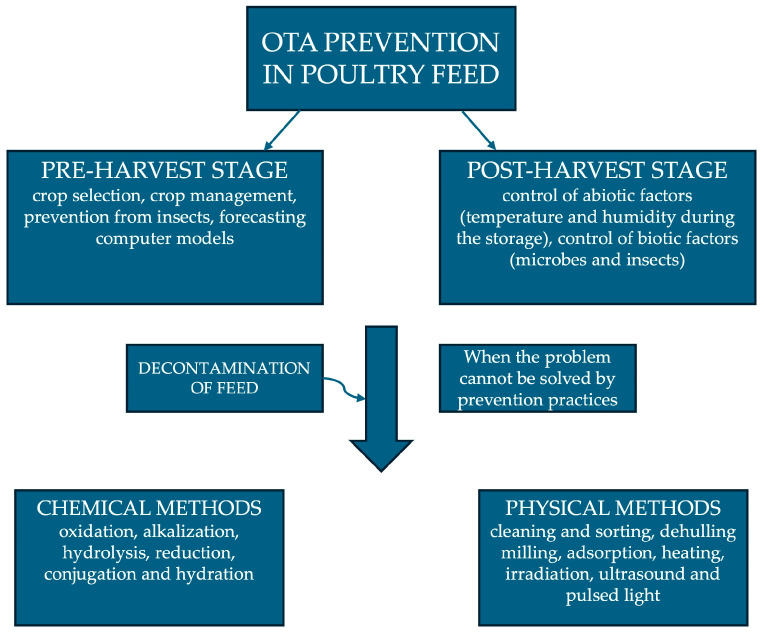
Prevention and decontamination methods from OTA in poultry feed.

The prevention and decontamination methods from OTA in feed described in this section are shown in Figure 2.

## 6. Conclusions

The main vehicle of OTA in poultry farming is feed. For this reason, the European Commission Recommendation (EU) 2016/1319 defines the guidance values for cereals and cereal products intended for feed materials. Compliance with these limits should be achieved to reduce the risk of contamination of human food by controls on raw material during pre-harvest and post-harvest phases and by using effective decontamination methods. Moreover, sampling of feed containers should be performed using an inspection model that allows for the collection of samples for analysis at different positions in the batch to avoid sampling errors due to the presence of mycotoxins in isolated parts.

Regarding OTA in poultry muscle and eggs, which are most used for commercial purposes, studies indicate on average low levels of contamination. However, more attention must be paid to the target organs of this mycotoxin, such as the kidney and liver, especially for specific segments of the European population that are significant consumers of locally produced avian specialties.

The post-mortem inspection of poultry species in slaughterhouses is subject to important limitations due to random sampling and the infrequent presence of the veterinary inspector, often replaced by auxiliary staff. These elements make it very difficult to identify any OTA-related lesions, which may often be internal and not visible, or non-specific. Therefore, effective risk management is crucial in livestock farming to prevent mycotoxins from contaminating animal feed and entering the food chain. In this context, ClassyFarm emerges as an innovative and strategic tool for assessing and controlling risks associated with animal production. It is an integrated system, developed in 2018 under guidance from the Italian Ministry of Health, that classifies farms based on risk analysis related to veterinary public health. This system assesses several critical areas such as biosecurity, animal welfare, health and production indicators, nutrition, antimicrobial drug use, and lesions identified at slaughterhouse. It currently supports evaluations for a range of livestock species and distinguishes classifications based on each species’ production type and farming method. By evaluating feeding practices and promoting biosecurity, ClassyFarm can identify and mitigate the risk of feed contamination by mycotoxins. Additionally, by enhancing traceability throughout the production chain, it facilitates targeted interventions and promotes operator training, helping to prevent mycotoxin presence and ensuring the safety of products of animal origin [96].

## Figures and Tables

**Table 1:** Distribution of OTA in tissues of avians fed with OTA-contaminated feed.

OTA Treatment	Sample Type	OTA Content	Method	Reference
Broiler chicks control/OTA-treated (0.1 mg kg^−1^ feed)/35 days	Broiler chick serum	Control: 0.2 ± 0.1 ng mL^−1^ Treated: 1.2 ± 0.4 μg kg^−1^	^2^ HPLC-FD	[63]
	Broiler chick liver	Control: ^1^ ND Treated: 1.9 ± 0.2 μg kg^−1^		
	Broiler chick kidney	Control: ^1^ ND Treated: 3.6 ± 0.9 μg kg^−1^		
	Broiler chick breast	Control: ^1^ ND		
	Broiler chick thigh	Control: ^1^ ND		
Laying Hens OTA-treated: 255–285 μg kg^−1^ feed	Laying hens kidney	13.65 ± 3.58 μg kg^−1^	^2^ HPLC-FD	[10]
	Laying hens liver	4.43 ± 0.64 μg kg^−1^		
	Laying hens other tissues	^1^ ND		
Laying Hens OTA-treated: 0.2 mg kg^−1^ feed/12 weeks	Laying hens kidney	2.47 ± 1.10 μg kg^−1^	^2^ HPLC-FD	[64]
	Laying hens muscle	0.31 ± 0.22 μg kg^−1^		
	Laying hens blood	1.06 ± 0.48 μg kg^−1^		
Broiler chicks OTA-treated: 0.5 mg feed/4 weeks	Broiler chick liver	^4^ MRC of OTA: 4 μg kg^−1^ after 7 days	^2^ HPLC-FD	[65]
		^4^ MRC of OTA: 1.4 μg kg^−1^ after 7 days	^3^ ELISA	
	Broiler chick kidney	^4^ MRC of OTA: 1.25 μg kg^−1^ after 7 days	^2^ HPLC-FD	
		^4^ MRC of OTA: 2.84 μg kg^−1^ after 7 days	^3^ ELISA	
	Broiler chick muscle	^4^ MRC of OTA: Thigh: 0.06 μg kg^−1^ after 21 days Breast: ^1^ ND	^2^ HPLC-FD	
		^4^ MRC of OTA: Thigh: 0.84 μg kg^−1^ after 28 days Breast: 0.84 μg kg^−1^ after 28 days	^3^ ELISA	
	Broiler chick plasma	^4^ MRC of OTA: 1.6 ng mL^−1^ after 14 days	^2^ HPLC-FD	
		^4^ MRC of OTA: 2.12 ng mL^−1^ after 14 days	^3^ ELISA	

^1^ ND: not detected; ^2^ HPLC-FD: high-performance liquid chromatography with fluorescence detector; ^3^ ELISA: enzyme-linked immunosorbent assay; ^4^ MRC: maximum residue content.

**Table 2:** Occurrence of OTA in meat and edible offal of avian species.

Country	Sample Type	Prevalence (%)	Mean of OTA Level (μg kg^−1^)	Range of OTA (μg kg^−1^)	Method	Reference
Denmark	Duck meat	11/19—57.8%	0.02	^1^ ND–0.09	^3^ HPLC ^10^ LOD: 0.02–0.03 μg kg^−1^	[67]
	Duck liver	4/7—57.1%	0.06	^1^ ND–0.16		
	Goose meat	5/12—41.6%	0.03	^1^ ND–0.10		
	Goose liver	4/12—33.33%	0.02	^1^ ND–0.06		
	Turkey meat	10/17—58.8%	0.02	^1^ ND–0.11		
	Turkey liver	3/17—17.6%	0.04	^1^ ND–0.28		
	Chicken meat	36/65—55.3%	0.03	^1^ ND–0.11		
Portugal	Chicken muscle	9/12—75%	^2^ NM	^2^ NM	^4^ HPLC-FD ^10^ LOD: 0.01 μg kg^−1^ ^11^ LOQ: 0.04 μg kg^−1^	[68]
	Turkey muscle	9/13—69%	0.02 ± 0.03	0.01–0.04		
Italy	Poultry kidney	0/120—0%	≤LOD	^2^ NM	^4^ HPLC-FD ^10^ LOD: 0.1 μg kg^−1^ ^11^ LOQ: 0.2 μg kg^−1^	[69]
Serbia	Chicken kidney	17/60—28.33%	0.51 ± 1.38	0.1—7.02	^4^ HPLC-FD ^10^ LOD: 0.2 μg kg^−1^ ^11^ LOQ: 0.3 μg kg^−1^	[70]
	Chicken liver	23/60—38.33%	0.58 ± 1.04	0.14—3.9		
	Chicken gizzard	16/60—26.6%	0.51 ± 1.75	0.25–9.94		
Italy	Hen kidney	^2^ NM	47 ± 3.03	^2^ NM	^4^ HPLC-FD	[14]
	Hen liver	^2^ NM	24 ± 1.92	^2^ NM		
Pakistan	Chicken broiler meat	16/39—41%	Wings: 1.39 ± 0.78 Chest: 0.28 ± 0.79 Legs: 1.12 ± 0.19 Liver: 2.21 ± 0.43	Wings: 0.06–2.50 Chest: 0.06–3.67 Legs: 0.06–2.12 Liver: 0.06–3.56	^4^ HPLC-FD ^10^ LOD: 0.06 μg/kg ^11^ LOQ: 0.18 μg/kg	[71]
	Chicken layers meat	22/45—48%	Wings: 1.45 ± 0.24 Chest: 0.81 ± 0.14 Legs: 1.59 ± 0.67 Liver: 2.41 ± 0.72	Wings: 0.06–3.90 Chest: 0.06–1.30 Legs: 0.06–2.10 Liver: 0.06–4.70		
	Domestic chicken meat	9/31—29%	Wings: ≤LOD Chest: ≤LOD Legs: ≤LOD Liver: 0.71 ± 0.40	Wings: ^1^ ND Chest: ^1^ ND Legs: ^1^ ND Liver: 0.06–2.40		
Jordan	Poultry thigh and leg	18/18—100%	2.160 ± 0.270	1.90 ± 0.14–2.98 ± 0.50	^5^ ELISA	[72]
	Poultry liver	18/18—100%	5.860 ± 0.390	4.06 ± 0.66–7.68 ± 0.12		
	Poultry gizzard	18/18—100%	2.070 ± 0.133	1.89 ± 0.07–2.26 ± 0.19		
	Poultry breast	12/18—66%	3.062 ± 0.300	2.81 ± 0.52–3.31 ± 0.18		
Jordan	Chicken meat	0/150—0%	≤LOD	^2^ NM	^6^ LC-TOF-MS/MS	[73]
	Chicken kidney	0/50—0%	≤LOD	^2^ NM		
	Chicken liver	0/50—0%	≤LOD	^2^ NM		
China	Chicken liver	1/5—20%	1.05	^2^ NM	^7^ LC-MS/MS ^10^ LOD: 0.15 μg kg^−1^ ^11^ LOQ: 0.46 μg kg^−1^	[74]
Iraq	Chicken meat	26/30—86.7%	1.982	0.149—4.10	^8^ HPLC-UV	[75]
	Chicken liver	17/30—57%	1.865	^1^ ND—4.702		
Cameroon	Chicken muscles	^2^ NM	1.4 ± 0.173	0.8–1.7	^5^ ELISA ^10^ LOD: 0.3–0.6 μg kg^−1^ ^11^ LOQ: 1–2 μg kg^−1^	[76]
	Chicken liver	^2^ NM	2.2667 ± 1	1–4.9		
Egypt	Broiler muscle (50 days old)	5/10—50%	5.94 ± 2.21	^2^ NM	^9^ TLC	[77]
	Broiler muscle (100 days old)	6/10—60%	7.41 ± 2.29	^2^ NM		
	Hen muscle (2 years old)	8/10—80%	9.66 ± 2.42	^2^ NM		
	Broiler fat (50 days old)	7/10—70%	9.51 ± 2.94	^2^ NM		
	Broiler fat (100 days Old)	7/10—70%	16.02 ± 4.08	^2^ NM		
	Hen fat (2 years old)	9/10—90%	17.39 ± 3.57	^2^ NM		
	Broiler gizzard (50 days old)	7/10—70%	8.45 ± 2.93	^2^ NM		
	Broiler gizzard (100 days old)	8/10—80%	15.69 ± 4.24	^2^ NM		
	Hen gizzard (2 years old)	9/10—90%	15.32 ± 3.33	^2^ NM		
	Broiler kidney (50 days old)	10/10—100%	12.90 ± 3.30	^2^ NM		
	Broiler kidney (100 days old)	10/10—100%	16.67 ± 3.86	^2^ NM		
	Hen kidney (2 years old)	10/10—100%	18.84 ± 3.30	^2^ NM		
	Broiler liver (50 days old)	8/10—80%	8.97 ± 2.83	^2^ NM		
	Broiler liver (100 days old)	9/10—90%	10.75 ± 3.07	^2^ NM		
	Hen liver (2 years old)	10/10—100%	14 ± 2.54	^2^ NM		

^1^ ND: not detected, ^2^ NM: not mentioned, ^3^ HPLC: high-performance liquid chromatography, ^4^ HPLC-FD: high-performance liquid chromatography with fluorescence detector, ^5^ ELISA: enzyme-linked immunosorbent assay, ^6^ LC-TOF-MS/MS: liquid chromatography/time-of-flight/tandem mass spectrometry, ^7^ LC-MS/MS: liquid chromatography coupled to tandem mass spectrometry, ^8^ HPLC-UV: high-performance liquid chromatography–ultraviolet, ^9^ TLC: thin layer chromatography, ^10^ LOD: limit of detection, ^11^ LOQ: limit of quantification.

**Table 3:** Occurrence of OTA in eggs.

OTA Treatment	OTA Content in Eggs	Method	Reference
Laying hens control/OTA- treated (0.1 mg kg^−1^ feed)/OTA-treated (0.2 mg kg^−1^ feed)/OTA-treated (1 mg kg^−1^ feed)/OTA-treated (2 mg kg^−1^ feed)/30 days	Control: ^1^ ND Treated (0.1 mg kg^−1^): ^1^ ND Treated (0.2 mg kg^−1^): ^1^ ND Treated (1 mg kg^−1^): ^1^ ND Treated (2 mg kg^−1^): ^1^ ND	^2^ HPLC-FD	[78]
Laying hens OTA-treated: 2 mg kg^−1^ feed/21 days	<^3^ LOD	^2^ HPLC-FD ^3^ LOD: 0.05 μg kg^−1^ ^4^ LOQ: 0.15 μg kg^−1^	[79]
Laying hens control/OTA- treated (3 mg kg^−1^ feed)/OTA-treated (5 mg kg^−1^ feed)/28 days	Control: ^1^ ND for all 28 days ^5^ MRC of OTA (3 mg kg^−1^): 4.857 ± 0.23 μg kg^−1^ after 13 days ^5^ MRC of OTA (5 mg kg^−1^): 7.396 ± 1.03 μg kg^−1^ after 21 days	^2^ HPLC-FD	[80]

^1^ ND: not detected, ^2^ HPLC-FD: high-performance liquid chromatography with fluorescence detector, ^3^ LOD: limit of detection, ^4^ LOQ: limit of quantification, ^5^ MRC: maximum residue content.

## Data Availability

Not applicable.

## References

[1] Chen Y., Chen J., Zhu Q., Wan J. (2022). Ochratoxin A in Dry-Cured Ham: OTA-Producing Fungi, Prevalence, Detection Methods, and Biocontrol Strategies—A Review. Toxins.

[2] Wang L., Hua X., Shi J., Jing N., Ji T., Lv B., Liu L., Chen Y. (2022). Ochratoxin A: Occurrence and Recent Advances in Detoxification. Toxicon.

[3] Imaoka T., Yang J., Wang L., McDonald M.G., Afsharinejad Z., Bammler T.K., Van Ness K., Yeung C.K., Rettie A.E., Himmelfarb J. (2020). Microphysiological System Modeling of Ochratoxin A-Associated Nephrotoxicity. Toxicology.

[4] Stoev S.D. (2022). New Evidences about the Carcinogenic Effects of Ochratoxin A and Possible Prevention by Target Feed Additives. Toxins.

[5] Stoev S.D. (2022). Studies on Teratogenic Effect of Ochratoxin A given via Mouldy Diet in Mice in Various Sensitive Periods of the Pregnancy and the Putative Protection of Phenylalanine. Toxicon.

[6] Gan F., Zhou Y., Hou L., Qian G., Chen X., Huang K. (2017). Ochratoxin A Induces Nephrotoxicity and Immunotoxicity through Different MAPK Signaling Pathways in PK15 Cells and Porcine Primary Splenocytes. Chemosphere.

[7] Khoi C.-S., Chen J.-H., Lin T.-Y., Chiang C.-K., Hung K.-Y. (2021). Ochratoxin A-Induced Nephrotoxicity: Up-to-Date Evidence. Int. J. Mol. Sci..

[8] World Health Organization, International Agency for Research on Cancer (1993). IARC Monographs on the Evaluation of Carcinogenic Risks to Humans: Some Naturally.

[9] Commission Regulation (EU) 2023/915 of 25 April 2023 on Maximum Levels for Certain Contaminants in Food and Repealing Regulation (EC) No 1881/2006. OJ L 119/103, 22.07.2024. https://eur-lex.europa.eu/legal-content/EN/TXT/?uri=CELEX%3A32023R0915.

[10] Bozzo G., Ceci E., Bonerba E., Desantis S., Tantillo G. (2008). Ochratoxin A in Laying Hens: High-Performance Liquid Chromatography Detection and Cytological and Histological Analysis of Target Tissues. J. Appl. Poult. Res..

[11] Ostry V., Malir F., Ruprich J. (2013). Producers and Important Dietary Sources of Ochratoxin A and Citrinin. Toxins.

[12] Schrenk D., Bodin L., Chipman J.K., del Mazo J., Grasl-Kraupp B., Hogstrand C., Hoogenboom L., Leblanc J., Nebbia C.S., Nielsen E. (2020). Risk Assessment of Ochratoxin A in Food. EFSA J..

[13] Petzinger E., Ziegler K. (2000). Ochratoxin A from a Toxicological Perspective. J. Vet. Pharmacol. Ther..

[14] Bozzo G., Pugliese N., Samarelli R., Schiavone A., Dimuccio M.M., Circella E., Bonerba E., Ceci E., Camarda A. (2023). Ochratoxin A and Aflatoxin B1 Detection in Laying Hens for Omega 3-Enriched Eggs Production. Agriculture.

[15] LNalle C.L., Angi A.H., Supit M.A.J., Ambarwati S. (2019). Aflatoxin and Ochratoxin A Contamination in Corn Grains and Sago (Putak Meal) from Different Sources for Poultry in West Timor, Indonesia. Int. J. Poult. Sci..

[16] Akinmusire O.O., El-Yuguda A.D., Musa J.A., Oyedele O.A., Sulyok M., Somorin Y.M., Ezekiel C.N., Krska R. (2019). Mycotoxins in Poultry Feed and Feed Ingredients in Nigeria. Mycotoxin Res..

[17] Zhai S., Zhu Y., Feng P., Li M., Wang W., Yang L., Yang Y. (2021). Ochratoxin A: Its Impact on Poultry Gut Health and Microbiota, an Overview. Poult. Sci..

[18] Ganesan A.R., Balasubramanian B., Park S., Jha R., Andretta I., Bakare A.G., Kim I.H. (2021). Ochratoxin A: Carryover from Animal Feed into Livestock and the Mitigation Strategies. Anim. Nutr..

[19] Mujahid H. (2019). Protective Effect of Yeast Sludge and Whey Powder against Ochratoxicosis in Broiler Chicks. Pak. Vet. J..

[20] Vlachou M., Pexara A., Solomakos N., Govaris A. (2022). Ochratoxin A in Slaughtered Pigs and Pork Products. Toxins.

[21] Vila-Donat P., Marín S., Sanchis V., Ramos A.J. (2018). A Review of the Mycotoxin Adsorbing Agents, with an Emphasis on Their Multi-Binding Capacity, for Animal Feed Decontamination. Food Chem. Toxicol..

[22] Malir F., Ostry V., Pfohl-Leszkowicz A., Malir J., Toman J. (2016). Ochratoxin A: 50 Years of Research. Toxins.

[23] Bozzo G., Ceci E., Bonerba E., Di Pinto A., Celano G.V., Tantillo G. (2013). Occurrences of Ochratoxin A in Slaughtered Wild Boar (Sus Scrofa). Ital. J. Food Saf..

[24] Commission Recommendation (EU) 2016/1319 of 29 July 2016 amending Recommendation 2006/576/EC as regards deoxynivalenol, zearalenone and ochratoxin A in pet food. O JL 208/58, 2.8.2016. https://eur-lex.europa.eu/legal-content/EN/TXT/HTML/?uri=CELEX:32016H1319.

[25] Alexander J., Autrup H., Bard D., Benford D., Carere A., Guido L.C., Cravedi J.P., Di Domenico A., Fanelli R., Fink-Gremmels J. (2006). Opinion of the Scientific Panel on Contaminants in the Food Chain [CONTAM] Related to Ochratoxin A in Food. EFSA J..

[26] Gong L., Zhu H., Li T., Ming G., Duan X., Wang J., Jiang Y. (2019). Molecular Signatures of Cytotoxic Effects in Human Embryonic Kidney 293 cells Treated with Single and Mixture of Ochratoxin A and Citrinin. Food Chem. Toxicol..

[27] Özcan Z., Gül G., Yaman I. (2015). Ochratoxin A Activates Opposing C-MET/PI3K/Akt and MAPK/ERK 1-2 Pathways in Human Proximal Tubule HK-2 Cells. Arch. Toxicol..

[28] Schieber M., Chandel N.S. (2014). ROS Function in Redox Signaling and Oxidative Stress. Curr. Biol..

[29] Kamp H.G., Eisenbrand G., Janzowski C., Kiossev J., Latendresse J.R., Schlatter J., Turesky R.J. (2005). Ochratoxin A Induces Oxidative DNA Damage in Liver and Kidney after Oral Dosing to Rats. Mol. Nutr. Food Res..

[30] Petrik J., Žanić-Grubišić T., Barišić K., Pepeljnjak S., Radić B., Ferenčić Ž., Čepelak I. (2003). Apoptosis and Oxidative Stress Induced by Ochratoxin A in Rat Kidney. Arch. Toxicol..

[31] Pyo M.C., Choi I.-G., Lee K.-W. (2021). Transcriptome Analysis Reveals the AhR, Smad2/3, and HIF-1α Pathways as the Mechanism of Ochratoxin A Toxicity in Kidney Cells. Toxins.

[32] Erikstein B.S., Hagland H.R., Nikolaisen J., Kulawiec M., Singh K.K., Gjertsen B.T., Tronstad K.J. (2010). Cellular Stress Induced by Resazurin Leads to Autophagy and Cell Death via Production of Reactive Oxygen Species and Mitochondrial Impairment. J. Cell Biochem..

[33] García-Pérez E., Ryu D., Kim H.-Y., Kim H.D., Lee H.J. (2021). Human Proximal Tubule Epithelial Cells (HK-2) as a Sensitive In Vitro System for Ochratoxin A Induced Oxidative Stress. Toxins.

[34] Lee H.J., Pyo M.C., Shin H.S., Ryu D., Lee K.-W. (2018). Renal Toxicity through AhR, PXR, and Nrf2 Signaling Pathway Activation of Ochratoxin A-Induced Oxidative Stress in Kidney Cells. Food Chem. Toxicol..

[35] Arbillaga L., Azqueta A., Ezpeleta O., Cerain A.L.d. (2006). Oxidative DNA Damage Induced by Ochratoxin A in the HK-2 Human Kidney Cell Line: Evidence of the Relationship with Cytotoxicity. Mutagenesis.

[36] Tozlovanu M., Canadas D., Pfohl-Leszkowicz A., Frenette C., Paugh R.J., Manderville R.A. (2012). Glutathione Conjugates of Ochratoxin a as Biomarkers of Exposure / Glutationski Konjugati Okratoksina A Kao Biomarkeri Izloženosti. Arh. Hig. Rada. Toksikol..

[37] Girgis S.M., Hassanane M.M., Kassem S.M., Nada S.A. (2023). Protective Role of Grape Seed Extract on Genotoxicity, Hepatic, and Renal Dysfunction Induced by Ochratoxin A in Rats. Int. J. Pharm. Biol. Sci. Arch..

[38] Ozawa S., Ojiro R., Tang Q., Zou X., Jin M., Yoshida T., Shibutani M. (2024). In Vitro and in Vivo Induction of Ochratoxin A Exposure-Related Micronucleus Formation in Rat Proximal Tubular Epithelial Cells and Expression Profiling of Chromosomal Instability-Related Genes. Food Chem. Toxicol..

[39] Thomadaki H., Scorilas A. (2006). *BCL2* Family of Apoptosis-Related Genes: Functions and Clinical Implications in Cancer. Crit Rev Clin. Lab. Sci..

[40] Wu X. (2002). BAX and BH3-Domain-Only Proteins in P53-Mediated Apoptosis. Front. Biosci..

[41] Shimizu S., Kanaseki T., Mizushima N., Mizuta T., Arakawa-Kobayashi S., Thompson C.B., Tsujimoto Y. (2004). Role of Bcl-2 Family Proteins in a Non-Apoptotic Programmed Cell Death Dependent on Autophagy Genes. Nat. Cell Biol..

[42] Ouyang L., Shi Z., Zhao S., Wang F.T., Zhou T.T., Liu B., Bao J.K. (2012). Programmed Cell Death Pathways in Cancer: A Review of Apoptosis, Autophagy and Programmed Necrosis. Cell Prolif..

[43] Song Y., Liu W., Zhao Y., Zang J., Gao H. (2021). Ochratoxin A Induces Human Kidney Tubular Epithelial Cell Apoptosis through Regulating Lipid Raft/PTEN/AKT Signaling Pathway. Environ. Toxicol..

[44] Khoi C.S., Lin Y.W., Chen J.H., Liu B.H., Lin T.Y., Hung K.Y., Chiang C.K. (2021). Selective Activation of Endoplasmic Reticulum Stress by Reactive-Oxygen-Species-Mediated Ochratoxin A-Induced Apoptosis in Tubular Epithelial Cells. Int. J. Mol. Sci..

[45] Darbuka E., Gürkaşlar C., Yaman I. (2021). Ochratoxin A Induces ERK1/2 Phosphorylation-Dependent Apoptosis through NF-ΚB/ERK Axis in Human Proximal Tubule HK-2 Cell Line. Toxicon.

[46] Chou X., Ding F., Zhang X., Ding X., Gao H., Wu Q. (2019). Sirtuin-1 Ameliorates Cadmium-Induced Endoplasmic Reticulum Stress and Pyroptosis through XBP-1s Deacetylation in Human Renal Tubular Epithelial Cells. Arch. Toxicol..

[47] Huang X., Feng Y., Xiong G., Whyte S., Duan J., Yang Y., Wang K., Yang S., Geng Y., Ou Y. (2019). Caspase-11, a Specific Sensor for Intracellular Lipopolysaccharide Recognition, Mediates the Non-Canonical Inflammatory Pathway of Pyroptosis. Cell Biosci..

[48] Rogers C., Erkes D.A., Nardone A., Aplin A.E., Fernandes-Alnemri T., Alnemri E.S. (2019). Gasdermin Pores Permeabilize Mitochondria to Augment Caspase-3 Activation during Apoptosis and Inflammasome Activation. Nat. Commun..

[49] Ding J., Wang K., Liu W., She Y., Sun Q., Shi J., Sun H., Wang D.-C., Shao F. (2016). Pore-Forming Activity and Structural Autoinhibition of the Gasdermin Family. Nature.

[50] Shi J., Zhao Y., Wang K., Shi X., Wang Y., Huang H., Zhuang Y., Cai T., Wang F., Shao F. (2015). Cleavage of GSDMD by Inflammatory Caspases Determines Pyroptotic Cell Death. Nature.

[51] Mao X., Li H., Ge L., Liu S., Hou L., Yue D., Du H., Pan C., Gan F., Liu Y. (2022). Selenomethionine Alleviated Ochratoxin A Induced Pyroptosis and Renal Fibrotic Factors Expressions in MDCK Cells. J. Biochem. Mol. Toxicol..

[52] Li H., Mao X., Liu K., Sun J., Li B., Malyar R.M., Liu D., Pan C., Gan F., Liu Y. (2021). Ochratoxin A Induces Nephrotoxicity in Vitro and in Vivo via Pyroptosis. Arch. Toxicol..

[53] Ozawa S., Ojiro R., Tang Q., Zou X., Woo G., Yoshida T., Shibutani M. (2023). Identification of Genes Showing Altered DNA Methylation and Gene Expression in the Renal Proximal Tubular Cells of Rats Treated with Ochratoxin A for 13 Weeks. J. Appl. Toxicol..

[54] Zhang B., Zhu L., Dai Y., Li H., Huang K., Luo Y., Xu W. (2020). An in Vitro Attempt at Precision Toxicology Reveals the Involvement of DNA Methylation Alteration in Ochratoxin A-Induced G0/G1 Phase Arrest. Epigenetics.

[55] Marin S., Ramos A.J., Cano-Sancho G., Sanchis V. (2013). Mycotoxins: Occurrence, Toxicology, and Exposure Assessment. Food Chem. Toxicol..

[56] Pavlovi N.M. (2013). Balkan Endemic Nephropathy--Current Status and Future Perspectives. Clin. Kidney. J..

[57] Yordanova P., Wilfried K., Tsolova S., Dimitrov P. (2010). Ochratoxin A and Β2-Microglobulin in BEN Patients and Controls. Toxins.

[58] Hmaissia Khlifa K., Ghali R., Mazigh C., Aouni Z., Machgoul S., Hedhili A. (2012). Ochratoxin A Levels in Human Serum and Foods from Nephropathy Patients in Tunisia: Where Are You Now?. Exp. Toxicol. Pathol..

[59] Abid S., Hassen W., Achour A., Skhiri H., Maaroufi K., Ellouz F., Creppy E., Bacha H. (2003). Ochratoxin A and Human Chronic Nephropathy in Tunisia: Is the Situation Endemic?. Hum. Exp. Toxicol..

[60] Sherazi S.T.H., Shar Z.H., Sumbal G.A., Tan E.T., Bhanger M.I., Kara H., Nizamani S.M. (2015). Occurrence of Ochratoxin A in Poultry Feeds and Feed Ingredients from Pakistan. Mycotoxin Res..

[61] Wang Q., Zhao Y., Chen P., Zeng R., Liang Y. (2022). Ochratoxin A and Zearalenone in Poultry Feed Samples from South China. J. Food Saf..

[62] Fareed G., Khan S., Anjum M., Ahmed N. (2014). Determination of Aflatoxin and Ochratoxin in Poultry Feed Ingredients and Finished Feed in Humid Semi-Tropical Environment. J. Adv. Vet. Anim. Res..

[63] Pozzo L., Cavallarin L., Antoniazzi S., Guerre P., Biasibetti E., Capucchio M.T., Schiavone A. (2013). Feeding a Diet Contaminated with Ochratoxin A for Broiler Chickens at the Maximum Level Recommended by the EU for Poultry Feeds (0.1 Mg/Kg). 2. Effects on Meat Quality, Oxidative Stress, Residues and Histological Traits. J. Anim. Physiol. Anim. Nutr..

[64] Zaghini A., Simioli M., Roncada P., Rizzi L. (2007). Effect of *Saccharomyces Cerevisiae* and Esterified Glucomannan on Residues of Ochratoxin A in Kidney, Muscle and Blood of Laying Hens. Ital. J. Anim. Sci..

[65] Biró K., Solti L., Barna-Vetró I., Bagó G., Glávits R., Szabó E., Fink-Gremmels J. (2002). Tissue Distribution of Ochratoxin A as Determined by HPLC and ELISA and Histopathological Effects in Chickens. Avian Pathol..

[66] Schrenk D., Bignami M., Bodin L., Chipman J.K., del Mazo J., Grasl-Kraupp B., Hogstrand C., Hoogenboom L., Leblanc J., Nielsen E. (2023). Risks for Animal Health Related to the Presence of Ochratoxin A (OTA) in Feed. EFSA J..

[67] Jørgensen K. (1998). Survey of Pork, Poultry, Coffee, Beer and Pulses for Ochratoxin A. Food. Addit. Contam..

[68] Guillamont E.M., Lino C.M., Baeta M.L., Pena A.S., Silveira M.I.N., Vinuesa J.M. (2005). A Comparative Study of Extraction Apparatus in HPLC Analysis of Ochratoxin A in Muscle. Anal. Bioanal. Chem..

[69] Guerrini A., Altafini A., Roncada P. (2020). Assessment of Ochratoxin A Exposure in Ornamental and Self-Consumption Backyard Chickens. Vet. Sci..

[70] Milićević D., Jovanović M., Matekalo-Sverak V., Radičević T., Petrović M.M., Lilić S. (2011). A Survey of Spontaneous Occurrence of Ochratoxin A Residues in Chicken Tissues and Concurrence With Histopathological Changes in Liver and Kidneys. J. Environ. Sci. Health C.

[71] Iqbal S.Z., Nisar S., Asi M.R., Jinap S. (2014). Natural Incidence of Aflatoxins, Ochratoxin A and Zearalenone in Chicken Meat and Eggs. Food Control.

[72] AL Khalail N.I. (2018). Prevalence of Ochratoxin A in Poultry Feed and Meat from Jordan. Pak. J. Biol. Sci..

[73] Alaboudi A.R., Osaili T.M., Otoum G. (2022). Quantification of Mycotoxin Residues in Domestic and Imported Chicken Muscle, Liver and Kidney in Jordan. Food Control.

[74] Cao X., Li X., Li J., Niu Y., Shi L., Fang Z., Zhang T., Ding H. (2018). Quantitative Determination of Carcinogenic Mycotoxins in Human and Animal Biological Matrices and Animal-Derived Foods Using Multi-Mycotoxin and Analyte-Specific High Performance Liquid Chromatography-Tandem Mass Spectrometric Methods. J. Chromatogr. B..

[75] Murad H.O.M. (2015). Levels of Ochratoxin A in Chicken Livers and Meat at Sulaimani City Markets. Int. J. Sci. Technol..

[76] Tatfo Keutchatang F.D.P., Tchuenchieu A.K., Nguegwouo E., Mouafo H.T., Bouelet Ntsama I.S., Kansci G., Medoua G.N. (2022). Occurrence of Total Aflatoxins, Aflatoxin B1, and Ochratoxin A in Chicken and Eggs in Some Cameroon Urban Areas and Population Dietary Exposure. J. Environ. Public Health.

[77] Elgazzar M.M. (1998). Ochratoxin a Residues in Meat and Edible Offals of Marketed Broilers and Hens. Assiut Vet. Med. J..

[78] Bozzo G., Bonerba E., Ceci E., Colao V., Tantillo G. (2011). Determination of Ochratoxin A in Eggs and Target Tissues of Experimentally Drugged Hens Using HPLC–FLD. Food Chem..

[79] Denli M., Blandon J.C., Guynot M.E., Salado S., Perez J.F. (2008). Efficacy of a New Ochratoxin-Binding Agent (OcraTox) to Counteract the Deleterious Effects of Ochratoxin A in Laying Hens. Poult. Sci..

[80] Hassan Z.U., Khan M.Z., Khan A., Javed I., Hussain Z. (2012). Effects of Individual and Combined Administration of Ochratoxin A and Aflatoxin B1 in Tissues and Eggs of White Leghorn Breeder Hens. J. Sci. Food Agric..

[81] Stoev S.D., Anguelov G., Ivanov I., Pavlov D. (2000). Influence of Ochratoxin A and an Extract of Artichoke on the Vaccinal Immunity and Health in Broiler Chicks. Exp. Toxicol. Pathol..

[82] Stoev S.D., Koynarsky V., Mantle P.G. (2002). Clinicomorphological Studies in Chicks Fed Ochratoxin A While Simultaneously Developing Coccidiosis. Vet. Res. Commun..

[83] Kumar A., Jindal N., Shukla C.L., Asrani R.K., Ledoux D.R., Rottinghaus G.E. (2004). Pathological Changes in Broiler Chickens Fed Ochratoxin A and Inoculated with *Escherichia coli*. Avian Pathol..

[84] Koynarski V., Stoev S., Grozeva N., Mirtcheva T., Daskalov H., Mitev J., Mantle P. (2007). Experimental Coccidiosis Provoked by Eimeria Acervulina in Chicks Simultaneously Fed on Ochratoxin A Contaminated Diet. Res. Vet. Sci..

[85] Hameed M.R., Khan M.Z., Khan A., Javed I. (2013). Ochratoxin Induced Pathological Alterations in Broiler Chicks: Effect of Dose and Duration. Pak. Vet. J..

[86] Vasiljević M., Marinković D., Milićević D., Pleadin J., Stefanović S., Trialović S., Raj J., Petrujkić B., Trialović J.N. (2021). Efficacy of a Modified Clinoptilolite Based Adsorbent in Reducing Detrimental Effects of Ochratoxin A in Laying Hens. Toxins.

[87] Hanif N.Q., Muhammad G., Siddique M., Khanum A., Ahmed T., Gadahai J.A., Kaukab G. (2008). Clinico-Pathomorphological, Serum Biochemical and Histological Studies in Broilers Fed Ochratoxin A and a Toxin Deactivator (Mycofix^®^ Plus). Br. Poult. Sci..

[88] Gupta S., Jindal N., Khokhar R.S., Asrani R.K., Ledoux D.R., Rottinghaus G.E. (2008). Individual and Combined Effects of Ochratoxin A and *Salmonella Enterica* Serovar Gallinarum Infection on Pathological Changes in Broiler Chickens. Avian Pathol..

[89] Elaroussi M.A., Mohamed F.R., Elgendy M.S., El Barkouky E.M., Abdou A.M., Hatab M.H. (2008). Ochratoxicosis in Broiler Chickens: Functional and Histological Changes in Target Organs. Int. J. Poult. Sci..

[90] Santin E., Paulillo A.C., Maiorka P.C., Alessi A.C., Krabbe E.L., Maiorka A. (2002). The Effects of Ochratoxin/Aluminosilicate Interaction on the Tissues and Humoral Immune Response of Broilers. Avian Pathol..

[91] Food and Agriculture Organization of the United Nations. Word Health Organization. Code of Practice for the Prevention and Reduction of Mycotoxin Contamination in Cereals. Codex Alimentarius International Food Standards 2003. https://www.fao.org/fao-who-codexalimentarius/sh-proxy/ru/?lnk=1&url=https%253A%252F%252Fworkspace.fao.org%252Fsites%252Fcodex%252FStandards%252FCXC%2B51-2003%252FCXC_051e.pdf.

[92] Peng W.-X., Marchal J.L.M., van der Poel A.F.B. (2018). Strategies to Prevent and Reduce Mycotoxins for Compound Feed Manufacturing. Anim. Feed Sci. Technol..

[93] Nada S., Nikola T., Bozidar U., Ilija D., Andreja R. (2022). Prevention and Practical Strategies to Control Mycotoxins in the Wheat and Maize Chain. Food Control.

[94] Amézqueta S., González-Peñas E., Murillo-Arbizu M., López de Cerain A. (2009). Ochratoxin A Decontamination: A Review. Food Control.

[95] Ding T., Cullen P.J., Yan W. (2022). Applications of Cold Plasma in Food Safety.

[96] The ClassyFarm System. https://www.classyfarm.it/index.php/en/what-en.

